# Myoelectric Arm Orthosis in Motor Learning-Based Therapy for Chronic Deficits After Stroke and Traumatic Brain Injury

**DOI:** 10.3389/fneur.2022.791144

**Published:** 2022-02-08

**Authors:** Svetlana Pundik, Jessica McCabe, Margaret Skelly, Ahlam Salameh, Jonathan Naft, Zhengyi Chen, Curtis Tatsuoka, Stefania Fatone

**Affiliations:** ^1^Brain Plasticity and NeuroRecovery Laboratory, Cleveland Functional Electrical Stimulation Center, Louis Stokes Cleveland Department of Veterans Affairs Medical Center, Cleveland, OH, United States; ^2^Department of Neurology, Case Western Reserve University School of Medicine, Cleveland, OH, United States; ^3^Geauga Rehabilitation Engineering, Cleveland, OH, United States; ^4^Department of Population and Quantitative Health Sciences, Case Western Reserve University, Cleveland, OH, United States; ^5^Department of Physical Medicine and Rehabilitation, Northwestern University Feinberg School of Medicine, Chicago, IL, United States

**Keywords:** stroke, traumatic brain injury, upper extremity, rehabilitation, robotics, exoskeleton device, orthotics

## Abstract

**Background:**

Technologies that enhance motor learning-based therapy and are clinically deployable may improve outcome for those with neurological deficits. The MyoPro™ is a customized myoelectric upper extremity orthosis that utilizes volitionally generated weak electromyographic signals from paretic muscles to assist movement of an impaired arm. Our purpose was to evaluate MyoPro as a tool for motor learning-based therapy for individuals with chronic upper limb weakness.

**Methods:**

This was a pilot study of thirteen individuals with chronic moderate/severe arm weakness due to either stroke (*n* = 7) or TBI (*n* = 6) who participated in a single group interventional study consisting of 2 phases. The in-clinic phase included 18 sessions (2x per week, 27hrs of face-to-face therapy) plus a home exercise program. The home phase included practice of the home exercise program. The study did not include a control group. Outcomes were collected at baseline and at weeks 3, 5, 7, 9, 12, 15, and 18. Statistics included mixed model regression analysis.

**Results:**

Statistically significant and clinically meaningful improvements were observed on Fugl-Meyer (+7.5 points). Gains were seen at week 3, increased further through the in-clinic phase and were maintained during the home phase. Statistically significant changes in Modified Ashworth Scale, Range of Motion, and Chedoke Arm and Hand Activity Inventory were seen early during the in-clinic phase. Orthotic and Prosthetic User's Survey demonstrated satisfaction with the device throughout study participation. Both stroke and TBI participants responded to the intervention.

**Conclusions:**

Use of MyoPro in motor learning-based therapy resulted in clinically significant gains with a relatively short duration of in-person treatment. Further studies are warranted.

**Clinical Trial Registration:**

www.ClinicalTrials.gov, identifier: NCT03215771.

## Introduction/Background

Chronic upper limb deficits after Traumatic Brain Injury (TBI) and stroke are prevalent and often severely debilitating (1). Approximately 17% of individuals with TBI (1) and upwards of 50% of individuals with stroke (2) do not fully recover upper limb function. These persistent upper limb deficits limit function and negatively impact quality of life (1, 3). Motor learning-based therapy (ML) utilizing high repetition and timely progression of task-oriented movements is one of the most effective neurorehabilitation methods available (4–6). However, implementation of ML principles is challenging because it requires a high dose of face-to-face therapy. As a result, many individuals do not fully recover and those most severely impaired see the least amount of functional return in response to interventions (7). Adjuvant technologies that facilitate ML and that are easily deployable in the current health care milieu are highly desirable, particularly for those with severe impairment. One such technology that warrants further study is the MyoPro (Myomo Inc, Cambridge MA).

The MyoPro is a customized myoelectrically controlled orthosis that utilizes volitionally generated electromyographic (EMG) signals from paretic muscles to assist movement of an individual's affected arm (8–10). The device completes the movement initiated by the user and encourages practice of coordinated movement (such as mitigating co-contraction of agonist and antagonist muscles). Both aspects are essential elements of ML (6). Previous studies of MyoPro in arm rehabilitation after stroke provide positive preliminary evidence for improvement in motor control (8–12), self-reported function (9), and perception of recovery (13). These studies offer an important framework for utilization of a myoelectrically controlled orthotic device in ML therapy, but do not fully evaluate it in a structured clinical program that includes both in-clinic and home use. As a result, gains were variable across studies, (8, 10–13) relatively modest (11, 12) or equivocal compared to task practice alone (12), and the outcomes lacked a broad spectrum of assessments (8, 13).

The purpose of the current study was to assess the use of MyoPro in ML for individuals with chronic upper limb motor impairment after either stroke or TBI. To address limitations in prior studies, we report longitudinal response to the study intervention, which included an elbow-hand version of the MyoPro, across the International Classification of Functioning (ICF) domains of impairment, function, and participation during both in-clinic therapy and home phases.

## Methods

### Overview of Study Design

**Figure 1:**
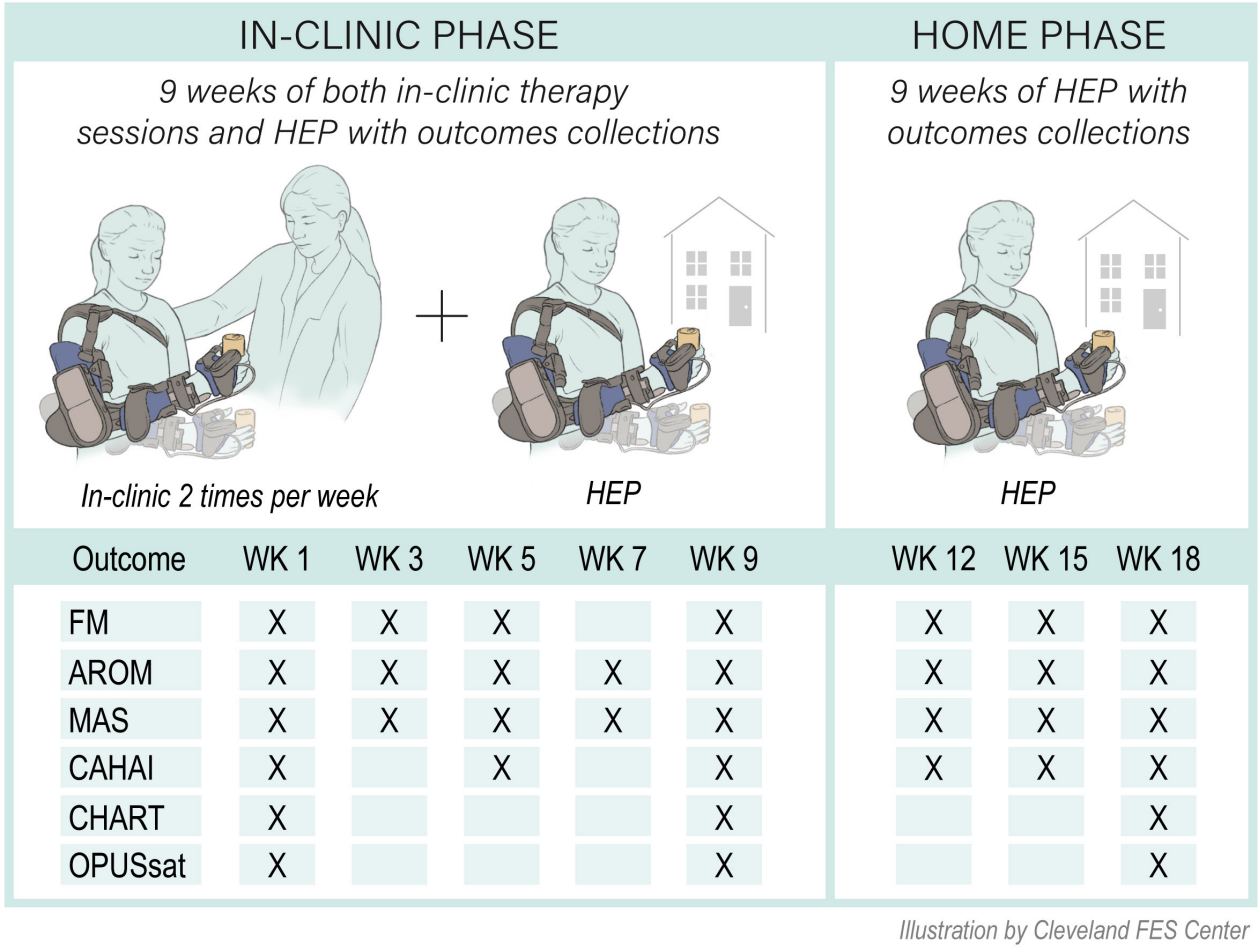
Study overview. FM, Fugl-Meyer for upper limb; MAS, Modified Ashworth Scale; ROM, active range of motion; CAHAI, Chedoke Arm and Hand Inventory; CHART, Craig Handicap Assessment and Rehabilitation Tool; OPUSsat, Orthotic and Prosthetic User's Survey Satisfaction Module; HEP, home exercise program; WK, week.

This was a prospective single arm mixed cohort interventional pilot study. After orthosis fitting/fabrication, individuals participated in two study phases: in-clinic therapy (9 weeks) and home phase (9 weeks) (Figure 1). Current clinical practice guidelines for outpatient rehabilitation for chronic stroke motor deficits suggests application of ML therapy at a minimum frequency of 45 minute sessions delivered 2 to 5 days per week for 8 weeks (14). Consistent with this, our in-clinic therapy phase consisted of 2 weekly sessions each lasting 1.5 h under the direction of a physical therapist trained in the application of motor learning-based upper limb intervention and use of the MyoPro. Sessions were divided into 45 min of training in the device and 45 min of training outside of the device. A customized home exercise program (HEP) was devised to complement in-clinic practice and consisted of in-device and out-of-device exercises tailored to the individual's needs. At the conclusion of the in-clinic phase, individuals transitioned to the home phase during which they were instructed to complete their customized HEP as prescribed. If needed, the HEP was adjusted/progressed during the testing sessions of the home phase. Outcome measures were collected during study participation as follows: at baseline (week 1); during the in-clinic phase (weeks 3, 5, 7, and 9); and during the home phase (weeks 12, 15, and 18) (Figure 1).

### Participant Selection

Participants were recruited by word of mouth and clinician referral within the medical center and surrounding local healthcare systems. The main inclusion criteria were as follows: first ever stroke or TBI ≥ 6 months prior to study entry; upper limb impairment that impeded function; medically stable; cognition sufficient to participate in training; caregiver support as needed; and ability to generate detectable EMG signals of the target muscles for training. Prior to study entry, participants provided informed consent or consent was provided by their legal guardian. The study was approved and monitored by the local Institutional Review Board of the medical center.

### Technology

**Figure 2:**
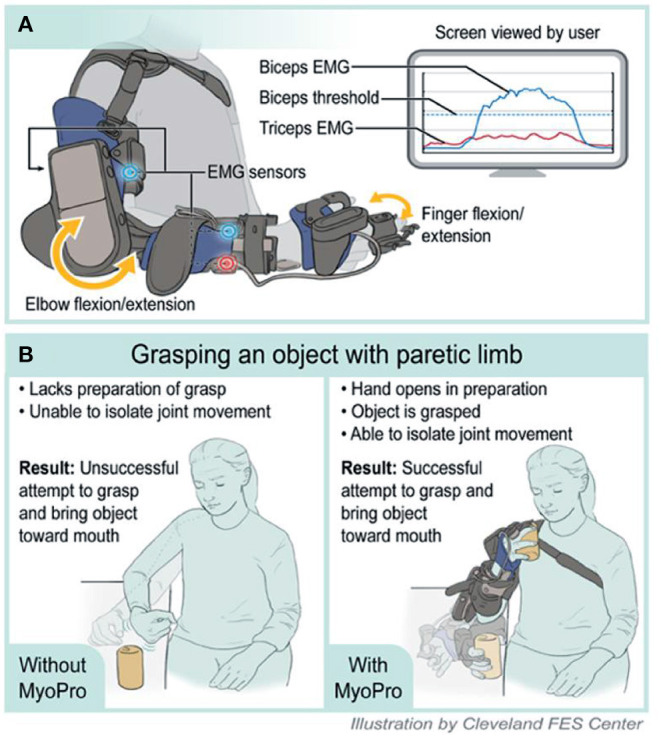
Illustration of MyoPro use and implementation in motor learning. **(A)** Device function. **(B)** Grasping an object with paretic limb. EMG, Electromyography.

The MyoPro is a commercially available, custom-fabricated myoelectric elbow-wrist-hand orthosis (Figure 2). EMG sensors placed over the biceps, triceps, finger flexors and finger extensors record the user's volitionally generated EMG (Figure 2A). When the EMG surpasses a threshold level set by a clinician, motors within the orthosis activate to assist with completion of the desired movement (Figure 2B).

### Orthosis Fitting/Fabrication and Monitoring

Each participant was fit with a custom MyoPro by a certified and licensed orthotist at the beginning of the study. After fitting, participants and their caregivers were trained in proper donning/doffing and operation of the device. Typically, within 2 sessions, individuals demonstrated competence with these tasks and took the MyoPro home from therapy sessions to practice during non-therapy days. Device fit was continuously monitored throughout study participation for signs of pressure or discomfort. If a participant noted any discomfort or persistent redness from device wear, adjustments were made by the treating therapist and/or orthotist.

### Intervention

Principles of motor learning that provided the theoretical framework for training included: movement practice as close to normal as possible, high repetition, progression of challenge, part vs. whole task practice, and knowledge of results (intrinsic/extrinsic feedback) (15). Based on these principles, treatment was customized to meet the specific capabilities of each participant and consisted of a combination of MyoPro training and ML therapy. The focus of the part or whole tasks included grasp/release, hand to mouth movements, forward reaching movements, bimanual tasks, and fine motor manipulation of objects. Training within the device was progressed using a hierarchy of challenge to increase complexity of movement (4, 5, 9). Initially, single muscles were trained to activate and relax. Training was progressed to include agonist/antagonist muscle training of a single joint; individual muscle activation of contiguous joints; and finally, agonist/antagonist coordination training of contiguous joints. For example, at commencement of elbow flexion training, individuals trained activation and relaxation of the biceps muscle in isolation. Then, training progressed to using EMG from both the biceps and triceps muscles simultaneously where to flex the elbow, the user had to activate the biceps while concomitantly relaxing the triceps. Similarly, for elbow extension, the user was trained to relax the biceps while activating the triceps. Training progressed from activation of single muscles of contiguous joints, such as activating the biceps while flexing the fingers in a hand to mouth movement to, finally, training more functionally complex movements. The goal was to produce activation/relaxation of agonist/antagonist pairs across contiguous joints such as bending the elbow and grasping an object, bringing it to the mouth, and then reaching out and placing it back on the table.

Motor learning-based exercises without MyoPro followed the same motor control hierarchy (4, 5), incorporating training of movements that could not be accomplished with the device, along with those that were trained with the device. Movement quality was carefully monitored, and training practice was incrementally progressed as soon as the participant demonstrated improved ability to perform a given task or movement component. Participants were instructed to perform the home program on non-clinic days and to increase repetition as they were able to tolerate. If a participant reported any discomfort related to exercise or activity at home, the therapist altered the HEP. After conclusion of the in-clinic phase, individuals transitioned to the home phase where they continued to utilize the custom HEP. They returned to the clinic at defined intervals for testing sessions and the treating therapist conducted weekly phone calls to maintain contact with participants and answer any questions. If any issues arose (i.e., need for device setting adjustments, home exercise progression), then an appointment was scheduled, and the participant was seen in the clinic.

### Outcome Measures

Data were collected according to the schedule in Figure 1. All measures were collected with the device doffed.

Fugl-Meyer (FM) for upper limb (the primary outcome measure) is one of the most widely used quantitative measures of motor impairment (16) with a Minimal Clinically Important Difference (MCID) of 5.25 points overall for upper limb function (16) in chronic stroke and 6 points in chronic TBI (17). Thirty-three items of movement coordination and reflex activity are scored with a 3-point Likert scale (0-66 points total) where higher scores represent less arm impairment. It has good intrarater (Intraclass Correlation Coefficient, ICC = 0.99) and interrater (ICC = 0.96) reliability for use with stroke patients (18).

Range of Motion (ROM) was assessed using standard clinical methods. Active ROM (AROM) and passive ROM (PROM) for elbow flexion/extension and wrist flexion/extension were measured using a goniometer (19). AROM was expressed as a percent of PROM, and a higher score was indicative of better performance.

Modified Ashworth Scale (MAS) was used to assess muscle tone. Using a 5-point scale, the clinician evaluates resistance to passive movement about a joint. A lower score represents less resistance to passive movement. The MAS has been widely used to quantify muscle tone following stroke. Interrater reliability of MAS for arm assessment has been reported as kappa = 0.92 or percent agreement = 97.4% (20). The following 9 muscle groups were assessed: shoulder internal rotators, biceps, triceps, pronators, supinators, wrist flexors, wrist extensors, finger flexors and finger extensors. Scores were then summed to give the overall MAS score (4).

Chedoke Arm and Hand Activity Inventory (CAHAI) was used to assess performance of activities of daily living (ADLs). This measure is suitable for populations with upper limb paresis (21) and consists of 13 functional tasks. Scoring is based on a 7-point scale (1 = unable; 7 = normal performance; maximum score is 91 points), where higher scores represent better performance of ADLs. MCID is 6.3 points in chronic stroke (22).

Craig Handicap Assessment and Rehabilitation Technique (CHART) is a life-role participation survey measuring the level of handicap using objectively observable behaviors in five dimensions: physical, social, cognitive, mobility, and occupation (23). Survey responses are combined in formulas for each domain. Although very social or active patients may score higher, the score in each domain is capped at 100 (total score range: 0–500). Higher scores represent better self-reported participation.

Orthotic and Prosthetic User's Survey satisfaction module (OPUSsat) is an 11-item patient-reported survey that assesses satisfaction with device using a 5-point Likert scale (24). Satisfaction with device is the sum of the scores (score range: 11–55), where higher scores indicate better satisfaction.

Orthosis utilization is the number of full and partial repetitions of elbow flexion/extension and hand open/close that were recorded by software within the MyoPro while the participant used the device. Purposeful movement cycles, defined as an EMG signal followed by 30° of motion and 1 s of no motion, were logged by the MyoPro motors.

Self-reported changes during study participation were recorded by study staff. Participants were queried at the beginning of intervention sessions and during testing visits of the home phase as to whether they experienced any changes in arm performance during their daily lives.

### Statistical Analysis

First, all variables and outcomes were examined univariately for association with injury type (stroke or TBI). Continuous variables were evaluated using Welch two sample *t*-tests, not assuming equal variance between the injury type groups; categorical variables were evaluated using Fisher's exact tests. Longitudinal linear mixed effects models were then fit to model the trajectory of outcomes through all time points. Two-sided significance level was set at 0.05 given only a single primary outcome was identified for this pilot study. For *post-hoc* analyses comparing changes from baseline with zero at different time points, *p*-values were adjusted for multiple testing using the Holm-Bonferroni correction. Longitudinal models included fixed effects for time (all the time points that data were collected during in-clinic and home phases), adjusted for corresponding baseline value and injury type, and random effects for subjects to account for within-subject correlation. Serial correlations among same subject outcomes were modeled. Covariance model selection was based on the model fit statistic**-**2 Res Log Likelihood. Analyses were performed using SAS Software (SAS Institute, Inc., Version 9.4, Cary, NC).

## Results

### Participant Characteristics, Baseline Scores

Sixteen individuals with chronic stroke (*n* = 8) or TBI (*n* = 8) were enrolled in the study. Thirteen individuals completed the study (stroke = 7; TBI = 6). Three participants withdrew due to issues unrelated to the study protocol. Participant's characteristics are provided in Table 1. The TBI cohort was younger than the stroke cohort (*p* < 0.001). Other baseline characteristics were not significantly different between the two injury type cohorts.

**Table 1:** Participant characteristics and device use at home.

**Subject**	**Age**	**Sex**	**Months post injury**	**Affected arm**	**Dominant arm**	**Injury type**	**Device use at home (hrs)**
1	74	male	118	right	right	Stroke	48.1
2	49	female	15	left	right	Stroke	2.3
3	82	male	48	right	right	Stroke	35.7
4	69	female	18	left	right	Stroke	12.8
5	59	female	23	left	right	Stroke	52.0
6	56	female	67	left	right	Stroke	52.7
7	69	female	20	right	left	Stroke	147.0
**Stroke Mean (SD) or count %**	**65.4 (11.4)**	**71.4 (% female)**	**44.1 (37.8)**	**42.9 (% right)**	**85.7 (% right)**		**50.1 (47.0)**
8	24	male	41	left	right	MVA	NC
9	25	male	89	left	right	GSW	653.1
10	52	female	344	left	left	MVA	69.6
11	43	female	354	right	right	MVA	50.2
12	29	male	29	left	right	GSW	1.9
13	27	female	125	left	right	GSW	35.1
**TBI Mean (SD) or count %**	**33.3 (11.5)**	**50.0 (% female)**	**163.7 (147.7)**	**16.7 (% right)**	**83.3 (% right)**		**162.0 (275.6)**
**Total cohort Mean (SD) or count %**	**50.6 (19.9)**	**61.5 (% female)**	**99.3 (116.8)**	**30.8 (% right)**	**84.6 (% right)**		**96.7 (179.3)**
**P-value for two-group comparison**	**<0.001**	**0.59**	**0.11**	**0.56**	**1.00**		**1.00**

*TBI, traumatic brain injury; MVA, motor vehicle accident; GSW, gunshot wound; NC, not collected*.

Table 2 lists baseline scores for all outcome measures by each participant and the means (SD) by injury type and for the whole cohort. The stroke cohort was more impaired than the TBI cohort at baseline according to AROM for wrist flexion/extension, and shoulder flexion (*p* = 0.009, 0.009 and 0.04, respectively). FM was marginally better but not statistically different in the TBI cohort (*p* = 0.07) compared with the stroke cohort. Baseline motor function (according to CAHAI) was significantly higher for the TBI cohort compared to the stroke cohort (*p* = 0.03).

**Table 2:** Baseline outcome measure scores.

**Subject**	**FM**	**MAS**	**Elbow extension AROM**	**Elbow flexion AROM**	**Shoulder abduction AROM**	**Shoulder flexion AROM**	**Wrist extension AROM**	**Wrist flexion AROM**	**CAHAI**	**CHART**	**OPUSsat**
1	32	9.5	55.2	55.2	100.0	57.1	18.2	18.2	30	331	70
2	23	10.5	58.1	58.1	22.2	33.3	0.0	0.0	17	271	NA
3	28	4	84.0	84.0	94.4	75.0	30.8	30.8	22	361.5	42
4	26	7.5	53.6	53.6	61.1	40.0	0.0	0.0	16	218	NA
5	26	11.5	39.3	39.3	72.2	39.1	0.0	0.0	25	497	47
6	19	5.5	15.4	15.4	72.2	43.5	0.0	0.0	16	244	66
7	16	11	0.0	0.0	61.1	42.3	0.0	0.0	14	329.8	86
**Stroke mean (SD)**	**24.3 (5.4)**	**8.5 (2.9)**	**43.6 (28.3)**	**43.6 (28.3)**	**69.1 (25.6)**	**47.2 (14.3)**	**7.0 (12.5)**	**7.0 (12.5)**	**20.0 (5.9)**	**321.8 (92.9)**	**62.2 (17.9)**
8	18	13.5	22.2	22.2	62.2	41.7	NA	NA	16	291.9	60
9	37	11	97.9	97.9	100.0	93.1	55.2	62.1	33	293	45
10	47	8	m	m	m	m	m	m	61	500	22
11	26	7	75.9	75.9	94.4	65.4	46.9	46.9	31	216	28
12	46	3.5	96.6	93.1	100.0	84.6	74.2	74.2	59	394	35
13	36	9	87.1	87.1	100.0	92.9	30.0	30.0	45	458	60
**TBI mean (SD)**	**35 (11.3)**	**8.7 (3.4)**	**75.9 (31.3)**	**75.2 (30.8)**	**91.3 (16.5)**	**75.5 (22.0)**	**51.6 (18.4)**	**53.3 (19.1)**	**40.8 (17.5)**	**358.8 (109.7)**	**41.7 (16.1)**
**Total cohort mean (SD)**	**29.2 (10.0)**	**8.6 (3.0)**	**57.1 (32.7)**	**56.8 (32.3)**	**78.3 (24.3)**	**59.0 (22.4)**	**23.2 (26.5)**	**23.8 (27.4)**	**29.6 (16.2)**	**338.9 (98.5)**	**51.0 (19.3)**
***P*** **-value for two-group comparison**	**0.07**	**0.93**	**0.10**	**0.11**	**0.10**	**0.04**	**0.009**	**0.009**	**0.03**	**0.53**	**0.08**

*FM, Fugl-Meyer for upper limb; MAS, Modified Ashworth Scale; AROM, active range of motion calculated as percent of passive range; CAHAI, Chedoke Arm and Hand Inventory; CHART, Craig Handicap Assessment and Rehabilitation Tool; OPUSsat, Orthotic and Prosthetic Users' Survey Satisfaction Module; m, missing*.

### Trajectory of Change Over the Course of the Study

**Figure 3:**
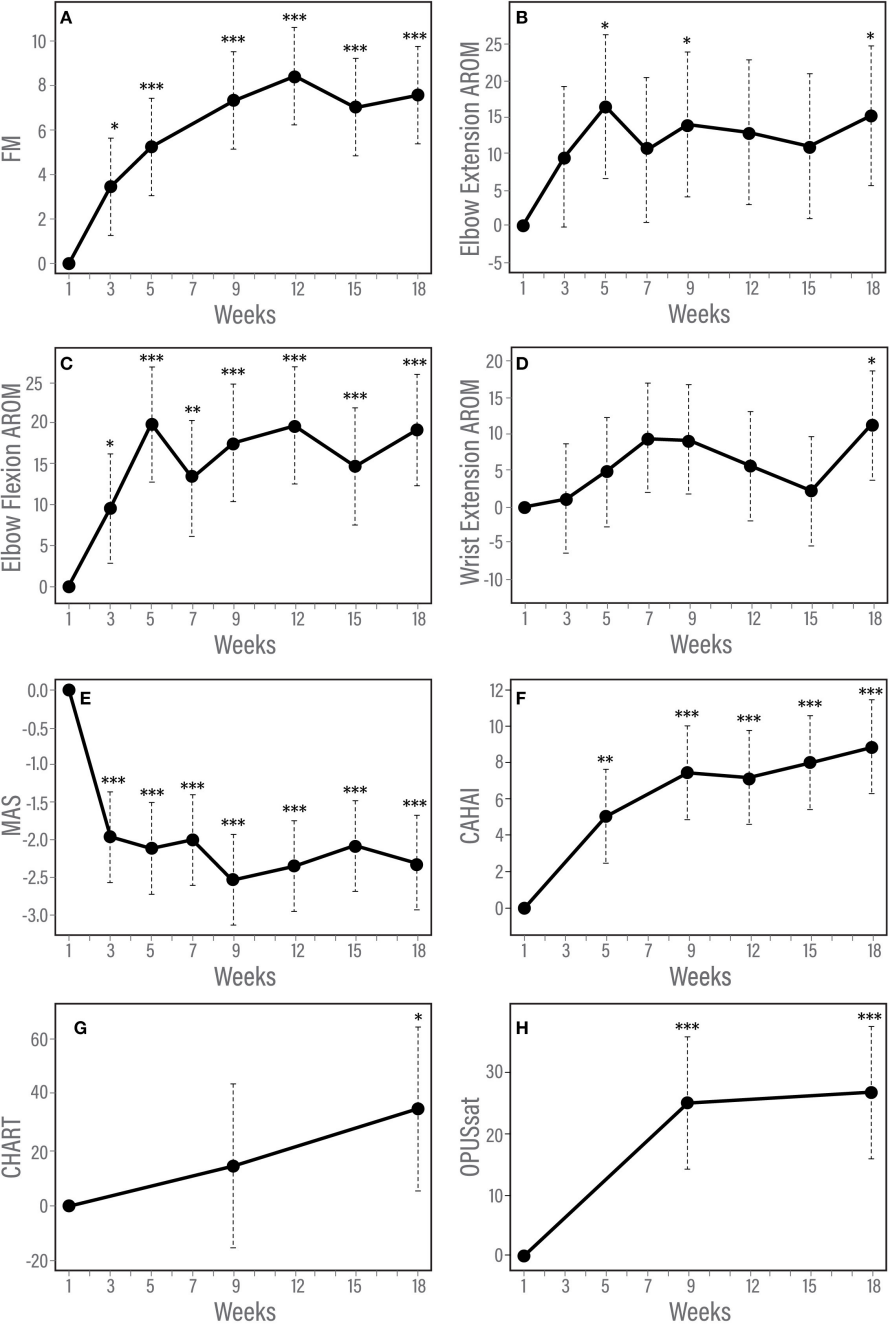
**(A–H)** Trajectory of changes in response to therapy according to measures of impairment, function and satisfaction. Results of longitudinal linear mixed effects model adjusted for baseline score and injury type. **(A)** Fugl-Meyer for upper limb, **(B)** Modified Ashworth Scale, **(C)** Elbow flexion active range of motion, **(D)** Elbow extension active range of motion, **(E)** Chedoke Arm and Hand Activity Inventory, and **(F)** Orthotic and Prosthetic Users's Survey satisfaction module. *p < 0.05; **p < 0.01; ***p < 0.001.

Changes from baseline for all outcome measures over the course of study participation are provided in Table 3. Figure 3 shows the results of longitudinal mixed model analysis adjusted for baseline score and injury type. *Post-hoc* analysis was used to assess for differences between the time points. Given sample size constraints, we only adjusted for injury type and baseline score, understanding that interpretation of results related to the injury type are intertwined with age differences.

**Table 3:** Outcome measure changes at different time points (mean (SD)).

		**Week 3**	**Week5**	**Week 7**	**Week 9**	**Week 12**	**Week 15**	**Week 18**
**FM**	**Stroke**	2.6 (3.4)	3.9 (4.1)		6.0 (3.9)	7.6 (3.8)	6.6 (3.9)	6.7 (4.4)
	**TBI**	4.3 (4.2)	6.7 (3.4)		8.7 (4.0)	9.2 (4.2)	7.3 (3.2)	8.3 (2.8)
	**All**	3.4 (3.7)	5.2 (4.0)		7.2 (4.0)	8.3 (3.9)	6.9 (3.5)	7.5 (3.7)
**MAS**	**Stroke**	−1.9 (0.9)	−1.7 (1.6)	−1.5 (1.5)	−2.1 (1.6)	−1.9 (0.8)	−1.6 (1.1)	−1.9 (1.1)
	**TBI**	−2.0 (0.9)	−2.5 (1.5)	−2.5 (1.7)	−3 (1.9)	−2.8 (1.4)	−2.5 (2.7)	−2.8 (1.4)
	**All**	−1.9 (0.9)	−2.1 (1.5)	−2.0 (1.6)	−2.5 (1.7)	−2.3 (1.2)	−2.0 (2.0)	−2.4 (1.3)
**Elbow ext. AROM**	**Stroke**	9.1 (10.6)	19.3 (16.1)	12.8 (8.7)	18.6 (15.4)	14.7 (23.1)	14.6 (17.2)	19.5 (26.7)
	**TBI**	11.3 (16.1)	6.5 (7.9)	1.4 (5.8)	0.8 (5.3)	4.7 (6.1)	−0.3 (7.1)	10.4 (23.3)
	**All**	10.0 (12.5)	14.7 (14.7)	8.7 (9.4)	12.1 (15.2)	11.1 (18.9)	9.2 (15.8)	15.8 (24.7)
**Elbow flex. AROM**	**Stroke**	9.1 (10.6)	25.4 (15.7)	17.9 (10.3)	25.0 (15.6)	26.2 (20.6)	21.5 (19.8)	26.2 (26.9)
	**TBI**	12.0 (15.5)	7.4 (7.1)	2.3 (4.2)	1.7 (4.6)	5.5 (5.4)	0.1 (5.4)	11.1 (22.9)
	**All**	10.3 (12.3)	18.9 (15.7)	12.2 (11.4)	16.5 (17)	18.7 (19.3)	13.7 (19)	19.9 (25.4)
**Shld. abd. AROM**	**Stroke**	−2.4 (18.1)	8.7 (16.9)	7.9 (17.2)	3.2 (13.6)	15.9 (21.6)	11.9 (26.1)	10.2 (28.8)
	**TBI**	1.4 (2.8)	1.4 (2.8)	1.4 (2.8)	1.4 (2.8)	1.4 (2.8)	1.4 (2.8)	−2.4 (8.9)
	**All**	−1.0 (14.2)	6.1 (13.7)	5.6 (13.8)	2.5 (10.6)	10.6 (18.3)	8.1 (21.0)	4.4 (22.2)
**Shld. flex. AROM**	**Stroke**	2.2 (12.2)	4.3 (15.8)	6.6 (11.1)	2.6 (8.7)	1.6 (12.8)	5.7 (9.7)	1.7 (11.8)
	**TBI**	9.5 (2.8)	6.5 (6.9)	8.6 (6.4)	6.7 (6.0)	6.4 (7.5)	2.2 (7.1)	0.1 (12.6)
	**All**	4.8 (10.3)	5.1 (12.9)	7.3 (9.3)	4.1 (7.8)	3.3 (11.0)	4.4 (8.7)	0.9 (11.6)
**Wrist ext. AROM**	**Stroke**	10.8 (13.8)	9.6 (10.4)	18.1 (15.2)	14.0 (8.0)	9.5 (10.6)	10.3 (11.9)	19.8 (13.4)
	**TBI**	−9.5 (9.9)	3.1 (5.3)	0.9 (9.7)	7.2 (8.5)	5.1 (12.9)	−6 (12.0)	5.0 (8.6)
	**All**	3.4 (15.7)	7.2 (9.2)	11.9 (15.6)	11.5 (8.5)	7.9 (11.1)	4.4 (14.0)	13.9 (13.6)
**Wrist flex. AROM**	**Stroke**	9.8 (14.3)	9.6 (10.4)	17.2 (16.2)	14.2 (9.8)	11.4 (9.0)	15.1 (13.6)	13.8 (12.4)
	**TBI**	−11.2 (6.6)	1.4 (5.7)	0.9 (9.7)	5.5 (9.8)	−1.6 (13.1)	−7.8 (14.6)	3.3 (9.8)
	**All**	2.2 (15.8)	6.6 (9.6)	11.3 (15.9)	11.0 (10.3)	6.7 (12.0)	6.8 (17.6)	9.6 (12.1)
**CAHAI**	**Stroke**		5.9 (3.8)		5.7 (3.1)	6.4 (3.7)	6 (2.2)	8.1 (4.7)
	**TBI**		4.2 (4.1)		9.5 (5.0)	8.2 (5.1)	10.3 (7.3)	9.7 (6.6)
	**All**		5.1 (3.9)		7.5 (4.4)	7.2 (4.3)	8 (5.5)	8.8 (5.5)
**OPUSsat**	**Stroke**				15.8 (23.7)			15.2 (24.5)
	**TBI**				32.7 (19.6)			36.3 (20.0)
	**All**				25.0 (22.2)			26.7 (23.7)
**CHART**	**Stroke**				23.1 (26.4)			55.5 (81.5)
	**TBI**				6.0 (30.5)			13.0 (26.8)
	**All**				15.2 (28.5)			35.9 (64.1)

*FM, Fugl-Meyer for upper limb; MAS, Modified Ashworth Scale; AROM, active range of motion calculated as percent of passive range; CAHAI, Chedoke Arm and Hand Inventory; CHART, Craig Handicap Assessment and Rehabilitation Tool; OPUSsat, Orthotic and Prosthetic Users' Survey Satisfaction Module; ext., extension; flex., flexion; shld., shoulder; abd., abduction*.

### FM for Upper Limb (FM)

**Figure 4:**
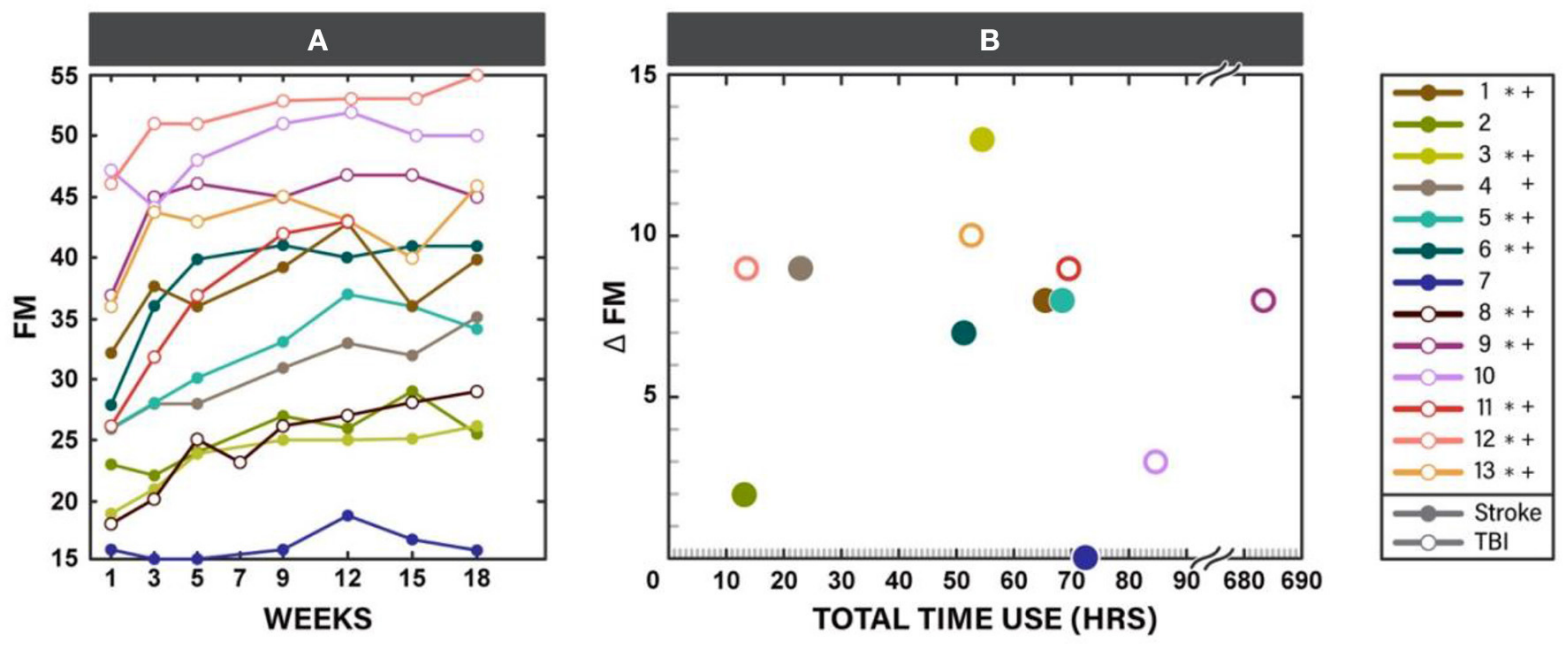
Fugl-Meyer for upper limb (FM) scores for each participant throughout the study participation **(A)** and scatter plot of change in FM at the end of the study (week 18) vs. total time of Myopro use **(B)**. * Marks Minimal Clinically Important Difference (MCID) for FM (5.25 points) at the end of the in-clinic phase (week 9); + marks MCID for FM at the end of the home phase (week 18).

The F test for overall differences among changes from baseline across time points was significant for FM (*F*_5, 60_ = 11.42, *p* < 0.001). Statistically significant changes *from baseline* were observed by week 3 of the in-clinic phase (*p* = 0.03). FM scores continued to improve after week 3 through the end of the clinic phase (week 9, *p* < 0.001) and were maintained during the home phase (weeks 12, 15, and 18, *p* < 0.001, Table 3, Figure 3A). Compared with week 5, statistically significant improvements were observed at weeks 9, 12 (*p* < 0.001) and 18 (*p* = 0.01). Neither baseline FM score nor injury type were associated with change in FM in response to therapy. A spaghetti plot of the individual participant data for the FM is provided in Figure 4A.

### MAS

The F test for overall differences among changes from baseline to all time points was significant for MAS (*F*_6, 71_ = 11.80, *p* < 0.001). MAS improved by week 3 (*p* < 0.001) and remained reduced through the end of the home phase (*p* < 0.001, Table 3, Figure 3B). Individuals with higher muscle tone at baseline demonstrated greater improvement in MAS score [estimate (95% CI) = −0.12 (−0.20, −0.04), *p* = 0.0023]. Injury type did not influence change in MAS in response to therapy.

### AROM

A significant F test for overall differences from baseline across time points was found only for wrist extension AROM (F_6, 59_ = 3.54, *P* = 0.0046). However, after adjustment for multiple testing, *post-hoc* pairwise analysis among time points did not show any significant results for wrist extension AROM. There was a statistically significant improvement from baseline in AROM for elbow flexion and elbow extension (Table 3, Figures 3C,D). Elbow flexion AROM improved by week 5 (*p* < 0.001) and was maintained through the end of the home phase (*p* < 0.001 for weeks 7, 9, 12, 15 and *p* = 0.0021 for week 18). Elbow extension AROM improved by week 3 (*p* = 0.048) and was maintained through the end of the in-clinic phase (*p* = 0.0063, 0.0091 and 0.018 for weeks 5, 7, and 9, respectively). There were no changes in AROM for shoulder abduction, shoulder flexion, wrist flexion or wrist extension (Table 3). For individuals with higher baseline AROM for elbow extension, their improvement in AROM was less [estimate (95% CI) = −0.23 (−0.46, −0.01), *p* = 0.045]. Similarly, individuals with higher baseline AROM for elbow flexion, their improvement in AROM was less [estimate (95% CI) = −0.36 (−0.42, −0.30), *p* < 0.001]. Injury type was not associated with change in ROM in response to therapy.

### CAHAI

The F test for overall differences among changes from baseline of all time points was significant for CAHAI (F_4, 48_ = 3.05, *P* = 0.026). Statistically significant improvement from baseline was noted for CAHAI by week 3 (*p* < 0.001) and scores continued to improve through the end of the home phase (*p* < 0.001, Table 3, Figure 3E). Compared with week 5, statistically significant improvements were observed at the end of home phase (*p* = 0.028). Neither baseline CAHAI score nor injury type influenced change in CAHAI in response to therapy.

### Chart

There were no significant changes in CHART (*p* = 0.10 and 0.074 for week 9 and 18, respectively, Table 3). Neither baseline CHART score nor injury type influenced change in CHART score in response to therapy.

### OPUSsat

A statistically significant improvement from baseline in OPUSsat was observed by the end of the in-clinic phase (week 9, *p* < 0.001) and was maintained at the end of the home phase (week 18, *p* < 0.001, Table 3, Figure 3F). Higher baseline score for OPUSsat was associated with lower improvement [estimate (95% CI) = −0.9 (−1.6, −0.2), *p* = 0.01]. Injury type did not influence change in OPUSsat score in response to therapy.

### Orthosis Utilization

Use of the device at home was similar during the two study phases (Table 4). There was no statistically significant difference between the TBI and stroke cohorts, although individual utilization varied (Table 1, last column). Change in FM plotted against the individual participant's orthosis utilization during study participation is provided in Figure 4B.

**Table 4:** Orthosis utilization for the whole cohort.

**In-clinic phase**	
	**Hours used in clinic**	**17.1 (5.4)**
	Number of days used in clinic^*^	**23.25 (2.5)**
	**Hours used at home**	**49.5 (86.4)**
	**Number of days used at home**	**27.0 (17.1)**
	**Hours/day at home**	**1.4 (1.5)**
	**Elbow Reps/day at home**	**85.8 (99.2)**
	**Hand Reps/day at home**	**194.5 (83.4)**
**Home phase**	
	**Hours**	**47.2 (93.8)**
	**Number of days**	**23.0 (23.3)**
	**Hours/day**	**1.5 (2.2)**
	**Elbow Reps/day**	**88.5 (91.5)**
	**Hand Reps/day**	**189.6 (127.1)**
**Total**	
	**Hours**	**113.9 (183.9)**
	**Elbow Reps**	**6847.3 (6785.4)**
	**Hand Reps**	**14493.8 (10066.0)**

* *Including testing sessions*.

### Self-Reported Changes During Study Participation

Participants reported several changes in their daily activities (Table 5). Improvements were reported with selfcare, feeding, home making, and mobility.

**Table 5:** Self-reported improvements in arm impairment and functional use.

**Categories**	**Proportion of participants reporting**	**Examples of self-reported changes**
Impairment	12/13	Increased grip strength Decreased stiffness
		Increased sensory awareness
		Emergence of finger movement/extension (e.g., pincer grasp)
Self-care	4/13	Ability to zip coat
		Holding toothbrush in hand instead of between legs
		Putting on deodorant
		Holding shower hose during bathing
Feeding	4/13	Using spoon
		Eating finger foods
		Drinking from a cup or bottle
		Peeling a grapefruit
Home making	9/13	Answering the phone
		Folding laundry
		Opening the refrigerator door
		Winding up an extension cord
		Carrying a paint can downstairs
		Opening a garbage bag
		Carrying a bag of grapes
		Getting a knife out of the drawer
		Washing dishes
		Feeding dog treats
Mobility	3/13	Maneuvering wheelchair more independently in the home environment
		Easier to walk up the stairs due to improved control of grasp on the railing
		Ability to maintain grasp on walker during walking

No serious device or study related events were observed and the device was well-tolerated by all participants.

## Discussion

This study provides encouraging results of using MyoPro, a myoelectrically controlled elbow-wrist-hand orthosis, as a tool in ML therapy for individuals with chronic arm deficits after stroke and TBI. Significant improvements were observed at the levels of impairment and function. Injury type and baseline impairment did not influence response to the study intervention. The MyoPro was well-tolerated, with no adverse events occurring during study participation.

### Clinically Meaningful Changes Utilizing a Short Face-to-Face Readily Deployable Therapy Protocol

High dose of rehabilitation is critical to improve motor performance (6), and it is likely that a dose that exceeds what is currently provided in clinical practice is needed (25). Furthermore, high dose must be combined with other key motor learning principles such as movement as close to normal as possible, knowledge of performance, and precise grading of progression to maximize its effectiveness (6). Studies have reported application of ML guided by these important principles (4, 5, 26). These studies demonstrated clinically significant improvement in individuals with moderate to severe chronic motor deficits after stroke in response to ML (4, 5, 26). However, these studies employed much higher dosing of in-person training compared to the current work. For example, after 90 h of face-to-face motor learning-based training, in a group of moderately impaired chronic stroke survivors (modified FM = 26 at baseline), median gain of FM was 6 points at the end of the intervention and 11 points at 6-month follow up (26). Following 300 h of in-person group motor-learning based training, there was a 9.7 point change on FM (4). At the 150 h midpoint, Daly et al. (4) reported a 5 point gain on FM, with additional improvement as therapy continued for another 150 h similar to the McCabe et al. study (4, 5). Thus, high doses of ML in-person therapy that carefully apply key motor learning principles can achieve clinically meaningful gains in motor abilities even in presumably plateaued individuals with chronic moderate to severe deficits. Unfortunately, the high-dose in-person therapeutic approach is challenging to implement in the current clinical milieu and alternative methods are desirable. Our protocol included only 27 h of face-to-face therapy, yet the results were similar to the studies with 90–300 h of in-person training (4, 5, 26). Though our cohort was more heterogenous in both injury type and level of baseline FM, it is encouraging that results were achieved with less face-to-face therapy hours.

Ability to achieve significant results with a clinically manageable amount of in-person contact hours is of great interest to the rehabilitation community. In fact, MyoPro has already been tried in clinical practice as a rehabilitation tool. In an uncontrolled observational study of MyoPro use in a group clinical setting, individual's chronic arm impairment after stroke demonstrated clinically meaningful improvement on FM of 9 points (8), however treatment application was highly varied across the individuals and home use was not tracked. The data collection and therapy delivery in this study aligned with those of general clinical practice; where data collection was not conducted in a systematic manner (i.e., collected at different timepoints based on the individual's performance) making group comparison challenging (8). Importantly, MyoPro appears to be easily deployed as a therapy tool in clinical practice.

We observed improvements across a spectrum of domains, i.e., impairment and function. The life role participation measure had a non-statistically significant trend toward improvement. The gain of 7.5 points on FM, an impairment measure, was in line with MCID for both stroke and TBI populations (17, 27). A statistically significant decrease in muscle tone according to MAS was observed in the first few weeks of in-clinic therapy and maintained for the duration of study participation. Though no definitive MCID value has been established for MAS in chronic stroke, a recent study of individuals undergoing rehabilitation attempted to interpret change on MAS in terms of clinical importance (28). It was found that a 0.76 point decrease in summed MAS score (consisting of elbow, wrist and finger flexors) was clinically significant in a group of 150 individuals followed longitudinally after stroke. In our cohort, we observed a decrease of 2 points in summed MAS of 9 muscle groups. While direct comparison with the clinical significance value cannot be made, it is encouraging that we observed a value exceeding that benchmark. At the level of function, statistically significant improvement in CAHAI was observed at the end of the in-clinic phase and continued into the home use phase. The mean improvement on CAHAI at the end of the in-clinic (7.4 points) and home phases (8.8 points) exceeded the established Minimal Detectable Change value of 6.3 points (22). On measures of satisfaction with device, there was statistically significant improvement in OPUSsat scores at the end of the in-clinic phase that was maintained at the end of the home phase. Improvement across the domains of impairment, function and satisfaction lends further support that this therapeutic approach is likely to be beneficial in the chronic phase of neurorecovery.

Our cohort was heterogeneous in level of impairment (baseline FM ranging 16–47) and included different types of injury (stroke and TBI). Furthermore, there was an age difference, with the TBI group younger than those with stroke. However, improvement was observed among individuals regardless of these factors. More treatment options exist for those with mild impairment after neurological injury (29) compared to those with severe deficits. Identification of clinically deployable approaches to treat the most severely impaired individuals are greatly needed (7). With this combination approach, individuals in the severe category demonstrated they could attain clinically meaningful improvement according to FM. Additionally, effective treatment methods that could be applied with different types of neurological diseases are desirable. We obtained similar results regardless of injury type (stroke vs. TBI) indicating in this preliminary study that the treatment approach was effective across diagnoses. Further studies are needed to test this approach in a controlled trial, but our current results are promising.

### Change in Motor Impairment and Relationship to Device Utilization

Practice patterns with the device varied within the cohort. Seven participants recorded orthosis utilization of approximately 50–70 h during study participation. Six out of these seven individuals had FM improvement that reached clinical significance. However, participants 4 and 12 also had clinically significant improvement on FM with only 10–25 h of orthosis utilization. Participant 4 presented with weakness, however early on during training she was able to regain the ability to activate her finger extensors outside of the device. Once she realized she was able to do this, she began using her hand in a more functional way and this may have contributed to her improvement on FM. Participant 12 had sufficient baseline motor function necessary to perform good quality motor task training outside of the orthosis and thus improved well on FM. Participant 9 demonstrated significantly higher device utilization than any of the other participants (over 600 h). While this participant had clinically significant improvement on FM, based on the relatively large amount of orthosis utilization it might be reasonable to expect change to be even greater. However, this participant reported wearing the device functionally throughout the day rather than continuously practicing the HEP with the device during the hours of wear. Lastly, participant 7 with severe motor impairment at baseline did not demonstrate change in FM despite having relatively high device utilization. The underlying reason why this participant did not improve on FM in the same manner as others is unknown, however it is possible that other impairments such as high tone may have impeded performance. In future studies, understanding predictors of response to intervention will allow for targeting of an intervention to those most likely to respond.

### Motor Learning-Based Therapy Principles Implemented Using Myoelectrically Controlled Device

Technological advancements within the rehabilitation field are occurring at an unprecedented pace and the options available to patients and clinicians have expanded greatly. It is incumbent upon the rehabilitation research community to identify technologies that are most likely to enhance current training methods.

MyoPro is the only commercially available assistive mechatronic upper limb device that is wearable, portable, and acts at 2 joints (29). Specific attributes of MyoPro make it an attractive technology to pair with ML. First, it is patient controlled. That is, the patient must generate volitional EMG for the device to assist with movement. Second, it is highly adjustable and portable. Settings can be incrementally adjusted to ensure adequate challenge during a given exercise and training is not constrained to activities in a stationary position (e.g., patients can wear the device and move about their environment to work on complex functional motor tasks). Training complexity can be progressed within the device. Initially, individuals may be able to control only a single muscle at a time. As they improve, the device can then be used to train coordination of multiple muscles to produce a functional movement. Third, it encourages patient-driven movement at high repetition with consistent repeatability. Motors within the device activate only when the individual's volitionally generated EMG reaches a threshold level; this then activates the device to assist the patient to complete a movement. The user can perform multiple repetitions of upper limb movement that may otherwise not be possible. This is particularly beneficial when trying to encourage high quality movement in the home setting for those with more severe deficits.

These special MyoPro attributes helped implement the three main motor-learning principles (repetition, close to normal movement, and task specific practice) and achieve clinically meaningful gains with only 27 h of a face-to-face therapy protocol. Specifically, the device facilitated implementation of progressive motor-learning principles even for those with severe impairment. Also, the device provided the ability to have meaningful practice of coordinated movement not only in clinic, but also at home. And, finally, the device motivated high-repetition practice. That is, participants experienced the movement when they volitionally attempted to activate a target muscle that otherwise would not occur (Figure 1). Therefore, the combination of therapist guided training with the device, motor learning during in-clinic practice, and the use of a complimentary individualized HEP using the device may have contributed to the current results approaching those of prior higher-dosed interventions (4, 5).

### Maintenance of Functional Gains

We observed maintenance of the achieved improvement through the home phase on all outcome measures. The maintenance of gains on FM at the end of in-clinic phase was similar to the maintenance of gains in other higher-dose studies (4). It is clinically assumed that maintenance of improvements is dependent on the level of impairment. In other words, individuals who are not too severe and able to incorporate their gains functionally in daily activity will maintain and continue to improve. The MyoPro provides meaningful continuous practice for those with severe deficits, which can be implemented in their own home setting.

## Limitations

While the results are encouraging, this study has several limitations. The sample size was small, no blinding was employed, and no comparison group was included. This curtails generalization of the results. However, we saw changes across impairment and function that deserve further study with a more rigorously controlled study design. Furthermore, this was a cohort of mixed diagnoses and heterogenous in terms of level of impairment. Regardless, both cohorts demonstrated response to the intervention.

## Conclusions

MyoPro might be a useful tool for ML in individuals with chronic stroke and TBI. Reduction in impairment, gains in function, and satisfaction with the device were observed in response to the intervention despite a lower dose of face-to-face therapy than prior studies. Further study using a randomized controlled design is warranted.

## Data Availability Statement

The raw data supporting the conclusions of this article will be made available by the authors, without undue reservation.

## Ethics Statement

This study involving human subjects was reviewed and approved by the Louis Stokes Cleveland VA Medical Center Institutional Review Board. The participants or their legal guardians provided written informed consent to participate in this study.

## Author Contributions

SP, SF, JM, and MS: conceptualization, methodology, and project administration. MS, JM, SP, SF, ZC, and CT: formal analysis. JM, MS, JN, and SP: investigation. SP, SF, JM, AS, and MS: data curation. SP and JM: writing—original draft preparation. JM, SP, SF, MS, AS, JN, ZC, and CT: writing—review and editing. SP and SF: supervision. SF: funding acquisition. All authors contributed to the article and approved the submitted version.

## Funding

The authors disclose the following financial support for the research, authorship and/or publication of this article. The U.S. Army Medical Research Acquisition Activity, 820 Chandler Street, Fort Detrick MD 21702-5014 is the awarding and administering acquisition office. This work was supported by the Office of Secretary of Defense for Health Affairs, through the Orthotics and Prosthetics Outcomes Research Program, Orthotics Outcomes Research Award under Award No. W81XWH-16-1-0733. Opinions, interpretations, conclusions, and recommendations are those of the author and are not necessarily endorsed by the Department of Defense. Myomo Inc. provided the devices and sponsored the device support.

## Conflict of Interest

The authors disclose the following potential conflict of interest with respect to the research, authorship and or publication of this article: JN is paid consultant with Myomo, Inc. The remaining authors declare that the research was conducted in the absence of any commercial or financial relationships that could be construed as a potential conflict of interest.

## Publisher's Note

All claims expressed in this article are solely those of the authors and do not necessarily represent those of their affiliated organizations, or those of the publisher, the editors and the reviewers. Any product that may be evaluated in this article, or claim that may be made by its manufacturer, is not guaranteed or endorsed by the publisher.

## Abbreviations

FM: Fugl-Meyer for upper limb
MAS: Modified Ashworth Scale
CAHAI: Chedoke Arm and Hand Activity Inventory
AROM: active range of motion
PROM: passive range of motion
CHART: Craig Handicap Assessment and Reporting Technique
OPUSsat: Orthotic and Prosthetic Users&#8217
Survey satisfaction module
TBI: traumatic brain injury.
